# Effect of Rice Flour Fermentation with *Lactobacillus spicheri* DSM 15429 on the Nutritional Features of Gluten-Free Muffins

**DOI:** 10.3390/foods9060822

**Published:** 2020-06-22

**Authors:** Maria Simona Chiş, Adriana Păucean, Simona Maria Man, Victorița Bonta, Anamaria Pop, Laura Stan, Bianca Vasilica Beldean (Tătar), Carmen Rodica Pop, Vlad Mureşan, Sevastiţa Muste

**Affiliations:** 1 Department of Food Engineering, Faculty of Food Science and Technology, University of Agricultural Sciences and Veterinary Medicine of Cluj-Napoca, 3-5 Mănăştur Street, 400372 Cluj-Napoca, Romania; simona.chis@usamvcluj.ro (M.S.C.); simona.man@usamvcluj.ro (S.M.M.); anamaria.pop@usamvcluj.ro (A.P.); bianca.beldean@usamvcluj.ro (B.V.B.); vlad.muresan@usamvcluj.ro (V.M.); sevastita.muste@usamvcluj.ro (S.M.); 2 Institute of Life Sciences, University of Agricultural Sciences and Veterinary Medicine Cluj-Napoca, 3-5 Mănăştur Street, 400372 Cluj-Napoca, Romania; victoritabonta@yahoo.com; 3 Department of Food Science, Faculty of Food Science and Technology, University of Agricultural Sciences and Veterinary Medicine of Cluj-Napoca, 3-5 Mănăştur Street, 400372 Cluj-Napoca, Romania; laurastan@usamvcluj.ro (L.S.); carmen-rodica.pop@usamvcluj.ro (C.R.P.)

**Keywords:** *Lactobacillus spicheri* DSM 15429, rice, bioactive compounds, muffins

## Abstract

*Lactobacillus Spicheri* DSM 15429 strain was used to ferment rice flour, aiming at exploiting its influence on the amino-acids, minerals, lactic acid, total phenols, and antioxidant activity of the rice sourdough and gluten-free muffins. Gluten-free muffins were prepared by using 15% rice sourdough fermented with the above strain of lactic acid bacteria and compared with rice spontaneous fermentation. Methods like LC-MS (Liquid chromatography–mass spectrometry), AA (atomic absorption), HPLC (High-performance liquid chromatography), Folin–Ciocalteu, and 1,1-Diphenyl-2-picrylhydrazyl radical scavenging activity (DPPH) were used to fulfill the aim of the study. The addition of rice sourdough fermented with LAB was reflected in the chemical composition of the final baked good, improving its amount on bioactive compounds such as amino acids, mineral bioavailability, total phenols, and antioxidant activity. Total phenols and antioxidant activity increased their amount by 70.53% and 73.70%, respectively, meanwhile, lactic acid, minerals, and amino-acids increased their values at least twice. Thus, rice fermented with *Lactobacilus spicheri* DSM 15429 strain could be a tool to further increase the nutritional value of gluten-free baked products.

## 1. Introduction

Consumer interest in healthy foods has increased in recent years, which has prompted recent research to find methods to produce healthy and functional foods. Among the myriad of healthy and functional foods available on the market, the category of gluten-free products is designed for humans diagnosed with celiac disease. Therefore, it must ensure all the nutritional, textural, and sensory requirements compared with the conventional gluten-containing products. The manufacture of gluten-free products represents a real challenge for the bakers, who should face technological problems due to the absence of gluten. According to the Codex Alimentarius, gluten is defined as “a protein fraction from wheat, rye, barley, oats, or their crossbred varieties and derivatives thereof, to which some persons are intolerant and that is insoluble in water and 0.5 mol/L NaCl” [1]. The technological role of gluten is the structure-forming ability in the viscoelastic matrix of the dough, which leads to certain sensory characteristics of the final product, like textural properties and overall appearance [2]. Its lack determines the unpleasant sensory feature of gluten-free products, an issue that influences negatively consumers’ acceptability [3]. Moreover, the celiac disease diet leads also to micronutrient and mineral deficiencies due to the malabsorption of the minerals, because of the atrophy of the intestinal villi [4]. All over the world, one of three people suffer from micronutrient deficiency, especially in Fe and Zn [4].

Rice (*Oryza sativa)* is considered as one of the significantly gluten-free vital food crops all over the world [5,6,7] being a unique crop due to its white color, soft taste, low sodium levels easily digestible carbohydrates and hypoallergenic properties [8]. This cereal crop has an important potential in the prevention of different diseases like cancer, chronic disorders due to its chemical composition rich in antioxidant compounds [9]. Therefore, its flour is an attractive raw material for manufacturing gluten-free foods [10].

Despite its numerous advantages, rice proteins have poor functional properties [11]. As already known, rice proteins cannot develop a viscoelastic network as wheat gluten does, which is responsible for retaining CO_2_ produced during the process of fermentation. Moreover, rice flour does not contain sufficient amounts of vitamins, minerals, and fiber which are important for a balanced diet of celiac patients due to the milling process [12,13,14]. For this reason, it is necessary to improve the quality of gluten-free products. Thus, new technological processes can be used, and one of them could be sourdough fermentation with lactic acid bacteria (LAB).

Rice is a staple food for almost 50% of the world’s population, being an important source of nutrients that could successfully be used as a substrate for the growth of LAB during the fermentation process [15,16,17,18]. Recently, fermentation became a trend to produce healthy foods from whole grain cereals. Industrial application of the biotechnology of fermentation for the production of gluten-free baked products is a promising innovation in the health foods industry [8].

Balli et al., (2019) [19] argue that cereal fermentation is useful for the release and the production of a greater amount of free phenolic compounds with respect to the unfermented substrate. The fermentation of the whole-rice flour might increase fiber solubility due to enzyme reactions on the cell wall structure [8,20]. Furthermore, according to Teleky et al., (2020) [21], LAB could influence through fermentation the proteolysis process, leading to the formation of bioactive peptides and the gluten degradation. LAB gluten degradation is also mentioned by Biscola et al., (2020) [22], who showed that the LAB could be involved in decreasing the food allergies. Maidana et al., (2020) [23] reported that LAB are involved in decreasing the amount of anti-nutritional factors and the value of the glycemic response. *Lactobacillus spicheri* strain was first described and isolated in 2004, from industrial rice sourdoughs by Meroth et al., (2004) [24]. Other authors such as Vogelmann et al., (2009) [15] have shown that *Lactobacillus spicheri* DSM 15429 strain can grow in a different vegetable matrix such as cassava, rye, and amaranth.

In this work, wholemeal rice flour was fermented with *Lactobacillus spicheri* DSM 15429 and the effects of fermentation on the nutritional and functional features were investigated. Gluten-free muffins fortified with rice flour sourdough were also characterized to point out their enhanced nutritional characteristics; these types of baked goods can be considered a valuable option for developing a healthy choice for celiac disease consumers. To the author’s knowledge, this is the first study aiming to investigate the nutritional influence of *Lactobacillus spicheri* DSM 15429 on rice flour fermentation and gluten-free muffins, respectively.

## 2. Materials and Methods

### 2.1. Materials

Rice wholemeal flour (RWF) originated from Germany, inulin, oatmeal, corn starch, baking powder, coconut butter, buckwheat flour, and maple syrup were purchased from Romanian specialized stores. The basic compositional parameters of RWF, determined according to Approved Methods of the American Association of Cereal Chemists, were: protein (8.5%), lipids (2.80%), moisture (10.40%), carbohydrates (78.0%), and ash (0.9%) [18].

The *Lactobacillus spicheri* DSM 15429 strain was acquired from Leibniz Institute-German Collection of Microorganism and Cell Cultures (Brunswick, Germany) and MRS (Man Rogosa Sharpe) was bought from Merck (Darmstadt, Germany). Analytical reagents and chemicals were purchased from Sigma Aldrich (St. Louis, MO, USA). All reagents were of analytical grade.

### 2.2. Microbial Starter Culture Preparation, Sourdough Preparation, and Muffins Formulation

*Lactobacillus spicheri* DSM 15429 (Lsp) was cultivated in MRS broth for 48 h at 30 °C. Afterward, the biomass was harvested by centrifugation (Eppendorf R 5804, Hamburg, Germany) at 2300× *g*, 10 min, 4 °C, washed and inoculated in the rice flour to reach an initial cell concentration of 10^7^ cfu/g, as previously described by Chiș et al., (2020) [18]. A spontaneous rice fermentation sourdough (OR) was used as a control and compared with sourdough with Lsp (SP) The sourdoughs were withdrawn at different fermentation times: 0, 4, 8, 12 and 24 h and coded as follows: SP 0H, SP 4H, SP 8H, SP 12H, and SP 24H for controlled fermentation and OR 0H, OR 4H, OR 8H, OR 12H and OR 24H for rice spontaneous fermentation, respectively.

**Figure 1:**
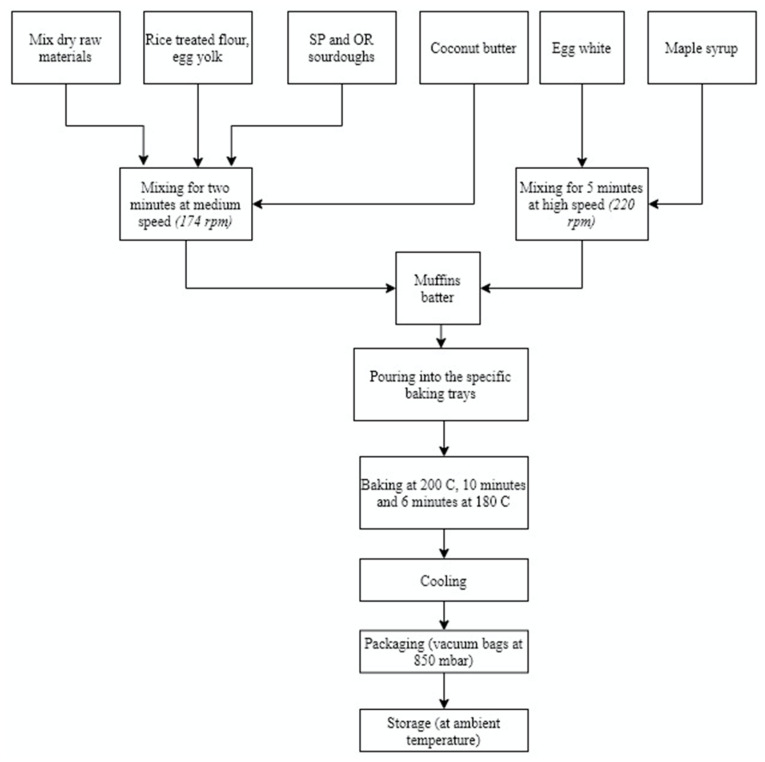
Flow diagram of muffin production.

The muffins formulation contained the following ingredients (*w*/*w*): mix dry raw materials (8% inulin, 10% oatmeal, 7% corn starch, 1.5% baking powder, and 8% buckwheat flour), treated RWF (32.5%), sourdough with Lsp (SP), and sourdough without Lsp (OR) 15%, eggs (8%), coconut butter (5%), and maple syrup (5%), as showed in Figure 1. Treated RWF was previously prepared according to Bourekoua et al., (2016) [25]. Shortly, RWF was mixed with tap water and hydrothermally treated at 65 °C, for 9 min. The final baked products were manufactured with SP and OR sourdough (illustrated in Figure 1) at different fermentation times: 0, 12, 24H and coded as follows: SPPF 0H, SPPF 12H, SPPF 24H and ORPF 0H, ORPF 12H, and ORPF 24H, respectively.

### 2.3. Amino-Acid Determination

Liquid chromatography–mass spectrometry (LC-MS) method was used to analyze the amino acids by using an LC-MS Shimadzu (Schimadzu Corporation, Kyoto, Japan) with electrospray ionization source, positive operating mode, according to the method described in our previous study by Bobiș et al., (2018) [26] and Chiș et al., (2018) [27]. An EZ:faast AAA-MS, 250 × 3.0 mm column (Phenomenex Torrance, Torrance, CA, USA) was used and the mobile phases were 10 mM ammonium formate in ultrapure water (A) and 10 mM ammonium formate in methanol (B) with a flow rate of 0.3 mL/min and an injection volume of 1 µL. The gradient started from 68% B to 83% B at 13 min. The percent of B decreased to 68% at 13.01 min and continued isocratically up to 17 min. The system was allowed to equilibrate for another 5 min at 68% B prior to the next analysis. The column temperature was 35 °C, the acquisition time: 33 min, while the detector voltage was set at 1.70 KV. A total amount of 0.25 g of each sample was mixed with 10 mL ultrapure water, sonicated for 30 min, and centrifugated (2300× *g*, 10 min) using Eppendorf 5804 centrifuge (Hamburg, Germany). After that, the samples were extracted and purified using the sample preparation method described in the EZ:faast Phenomenex kit, which include three steps: solid phase extraction, derivatization and liquid-liquid extraction. The values were expressed as mg/100 g product.

### 2.4. Minerals Content

Analysis of Macro and Microelements

Macro and microelements were determined by atomic absorption spectrophotometry (AAS), according to Păucean et al., (2018) [28] and Mihoc et al., (2012) [29]. Briefly, 3 g of sample was burned during 10 h at 550 °C in the furnace (Nabertherm B150, Lilienthal, Germany). The ash was moved to a volumetric flask of 20 mL, dissolved in HCl 20% (*w*/*v*) and the macroelements and microelements were analyzed by AAS (Varian 220 FAA equipment, Germany). Method detection limits (LOD mg/L) for analyzed elements were: 0.02 mg/100 g for Mg and K; 0.06 mg/100 g for Fe; 0.03 mg/100 g for Ca, Zn, Cu, Cr, and Mn.

The formula used to express the amount of macro and microelements is shown in Equation (1):

(1) $$E=\frac{C\cdot V\cdot 1000}{M\cdot 1000\;*}$$

where: *E*—the name of the element; *C*—the quantity taken from the standard curve, expressed in micrograms; *V*—total volume of the sample solution (mL); *M*—the amount of sample expressed in grams; 1000—content reporting factor at 1000 g; 1000 *—the conversion factor of micrograms into milligrams. All samples were analyzed in triplicate.

### 2.5. Lactic Acid Determination

High-performance liquid chromatography analysis HPLC-UV detection (Agilent Technologies 1200 Series, USA; Kyoto, Japan) was used to determine the lactic acid content, according to the method published by Păucean et al., (2013) [30], and Chiș et al., (2020) [18]. Briefly, 2 g of samples were homogenized with 5 mL of bi-distilled water, sonicated and centrifuged at 2300× *g*, for 15 min, using Eppendorf 5804 centrifuge (Eppendorf, Hamburg, Germany). The supernatant was filtered through a 0.22 µm filter and used for HPLC analysis. An Acclaim OA column, 5 µm, 4 × 250 mm Dionex was used for the lactic acid separation with a NaH_2_PO_4_ 50 mM mobile phrase (pH = 2.8) with an isocratic elution at a flow rate of 0.6 mL/min. The detection of lactic acid was set at a wavelength of λ = 210 nm. A standard curve of lactic acid was use to establish the samples concentration in lactic acid (y = 16.937x + 149.26, R^2^ = 0.9971, with 0.85 μg/mL minimum concentration and 85 μg/mL maximum concentration).

### 2.6. Total Phenols Assay by Folin–Ciocalteau Reagent

To determine the total phenolic (TF) content and antioxidant activity, extracts were prepared according to Bunea et al., (2011) [31] and Păucean et al., (2019) [32], as follows: 1 g of sample was mixed with 100 mL acidified methanol (85:15 v:v, MeOH:HCl) and stirred for 24 h by using a magnetic stirrer (Velp magnetic stirrer, Usmate (MB)–Italy. The obtained solutions were dried at 40 °C by using a vacuum rotary evaporator (Laborota 4010 digital rotary evaporator, Heidolph Instruments GmbH & Co. KG, Schwabach, Germany) and recovered in 10 mL methanol (99.9% purity). All samples extracts were filtered through 0.45 μm nylon filter (Millipore, Merck KGaA, Darmstadt, Germany).

The phenolic content was evaluated by Folin Ciocalteu colorimetric method previously published by Dordević et al., (2010) [33] and Păucean et al., (2019) [31] with slight modification. The reaction mixture contained 100 μL extract, 500 μL Folin–Ciocalteu reagent and 6 mL of distilled water. After that, 2 mL of 15% Na_2_CO_3_ were added and the solution was brought up to 10 mL with distilled water. The solution was kept in the dark and the absorbance was determined at 765 nm by using a UV/visible spectrophotometer Schimadzu 1700 (Shimadzu Corporation, Kyoto, Japan). The concentration of TF was expressed as milligrams of gallic acid equivalent (GAE) per 100 g product.

### 2.7. Antioxidant Capacity by DPPH Assay

The free radical scavenging capacity of the sourdough extracts was analyzed using the stable 1,1-Diphenyl-2-picrylhydrazyl (DPPH) radical, as described by Dordević et al., (2010) [33] and Chiș et al., (2018) [34]. Shortly, 0.1 mL of each methanolic extract was mixed with 3.9 mL DPPH, left in the dark at room temperature for 30 min. The absorbance of the resulting solution was read at 515 nm on a Schimadzu 1700 UV/visible spectrophotometer (Shimadzu Scientific Instruments, Kyoto, Japan). The radical scavenging activity was calculated as shown in Equation (2):

(2) $$RSA\left[\%\right]=\frac{{Abs}_{DPPH}*{Abs}^{Sample}}{{Abs}_{DPPH}}*100$$

where: *Abs^DPPH^* = absorbance of DPPH solution; *Abs_Sample_* = absorbance of the sample.

### 2.8. pH Determination

The pH values during 24 h fermentation of OR and SP were analyzed according to Păucean et al., (2019) [32], using a WTW pH-meter (Hanna Instruments, Vȯhringen, Germany). The samples were analyzed at 0, 4, 8, 12, and 24 h of fermentation.

### 2.9. Microbiological Analysis of Gluten-Free Muffins

#### Total Number of Yeast and Molds Determination

The determination was performed based on the method described by SR ISO 21527-2/2008 standard [35], by Antoniewska et al., (2018) [36] and Naghy et al., (2017) [37]. Briefly, 5 g of each sample were mixed with 45 mL of 0.1% peptone water diluent (Oxoid Ltd., Basingstoke, Hampshire, England) in a stomacher (Bag Mixer 100 MiniMix, Interscience, St. Nom, France). Further decimal dilution (10^−2^) was prepared in 0.1% peptone water diluent. All dilutions were inoculated in duplicate. 100 µL of the initial dilution (10^−1^) were aseptically transferred to a Petri dish containing Dichloran-Glycerol (DG18) agar (Oxoid Ltd., Basingstoke, Hampshire, England) using a sterile pipette and spread immediately with a Drigalski-spatula.

The procedure was repeated with further decimal dilution (10^−2^). The inoculated dishes were inverted and incubated at 25 °C for 5 days. After incubation, the visible colonies on selected plates (less than 150 colonies) were counted using a colony counter Colony Star 8500, (Funke-Dr. N. Gerber Labortechnik, Berlin, Germany).

### 2.10. Statistical Analysis

Data were analyzed using Duncan multiple comparison test by performing SPSS version 19 software (IBM Corp., Armonk, NY, USA). The results of three independent (*n* = 3) assays performed with replicates each were expressed as means ± standard deviations. Pearson correlation was computed on Minitab 19.1 (Minitab Inc., State College, PA, USA) at 95 confidence level.

## 3. Results

### 3.1. Amino Acids, Mineral, Total Phenols Content, and Radical Scavenging Activity of RWF

#### 3.1.1. Amino-Acids Content of the RWF

A total number of 21 individual amino-acids from RWF, sourdoughs, and the final baked muffins (OR PF and SP PF) were identified and grouped in five groups, as follows: acid, basic, aliphatic, aromatic, γ-aminobutyric, according to Kati et al., (2004) [38]. The acid group was formed by glutamine, asparagine, glutamic and aspartic acids, the basic one by histidine, lysine, ornithine, and arginine, the aromatic by tryptophan, tyrosine and phenylalanine, the aliphatic by prolamine, isoleucine, leucine, valine, alanine, and glycine) and ɣ-aminobutyric group by proline, threonine, serine, and ɣ-aminobutyric acid (GABA).

The amino-acids from the RWF are presented in Table 1. Aspartic acid was the representative amino-acid from the acid group with a total amount of 37.06 mg/100 g fresh weight (f.w.). Arginine with 11.12 mg/100 g f.w. was the highest amino-acid from the basic group. Tyrosine, alanine, and γ-aminobutyric acid were the amino-acids with the highest amount from aromatic, aliphatic, and γ-aminobutyric groups.

Lysine, which is the first limiting amino-acid among cereals’ amino acids, was detected in a higher content in RWF. The higher lysine content could be explained due to the rice bran albumin fraction, which contains higher amounts of lysine [39].

#### 3.1.2. Mineral Content of RWF

Rice is an important vegetable source of essential minerals being rich in calcium and magnesium [40,41]. The initial mineral content of rice flour (Ca, Mg, Fe, Cu, Zn, and Mn) displayed in Table 2 agrees with other findings [40,41,42], except K. The potassium content reported by others authors were lower than the values obtained in this study, e.g., 146 mg/100 g f.w. reported by Amagliani et al., (2017) [39], 141.5 mg/100 g by Mbatchou et al., (2013) [43] and 127 mg/100 g by Heinemann et al., (2015) [44].

The difference may be due to the type of rice, the mineral content of the soil, the water used for its cultivation, the environmental factors, and the degree extraction of the flour [42].

#### 3.1.3. Total Phenols and Radical Scavenging Activity of RWF

Rice is a widely consumed staple food with a rich chemical composition in phenolic acids like ferulic, p-Coumaric, isoferulic, syringic, vanillic, sinapic, caffeic, p-Hydroxybenzoic and protocatechuic acid of which ferulic acid is the most representative one [45]. In the present study, the RWF total phenols content was 150 ± 0.31 µg GAE/100 g dry weight (d.w.). This value is close to the value reported by Sakač et al. [46] who determined a value of 108 ± 8.1 µg GAE/100 g d.w.

Regarding the radical scavenging activity of rice flour the result obtained of 50% RSA is different from that determined by Sakač et al. [43] as 31%, but closer to the value found by Butsat et al. [47] for milled rice of 39% RSA. Gorinstein et al. [48] reported a value of rice milled flour of 20% RSA and 79% RSA for bran rice, respectively.

In the present study, the flour used to manufacture the sourdough was wholemeal correlated with the fact that the literature reported a value of bran rice of 85.9% RSA [47] and 79% [48] respectively, the value obtained could be justified. The high content of the bran rice in RSA is due to the phenolic acids, γ-oryzanols, and tocopherols content. The idea that rice bran has higher antioxidant activity due to the presence of the feluric acid, p-Coumaric acid, and sinapic acid is also supported by Fitriani et al. [49] and Lloyd et al. [50].

### 3.2. Amino-Acids, Mineral, Total Phenols Content, and Radical Scavenging Activity of OR and SP Sourdoughs

#### 3.2.1. Amino-Acids Content of SP and OR Sourdoughs

**Figure 2:**
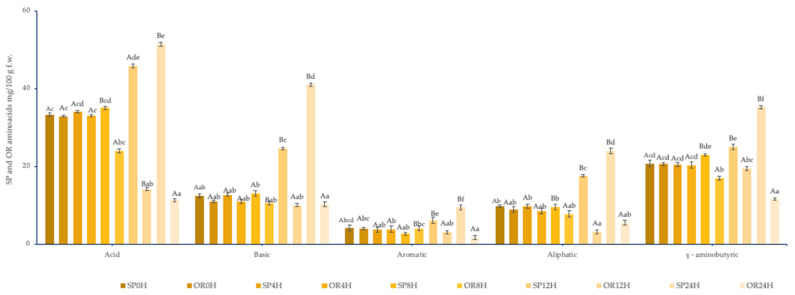
Amino acids content of sourdoughs with *L. spicheri* DSM 15429 (SP) and without *L. spicheri* DSM 15429 (OR) at different fermentation times: 0, 4, 8, 12, 24H, (Values not sharing the same small letters indicate the significant difference between OR and SP at different moments; 0, 4, 8, 12, 24H.; Values not sharing the same capital letters indicate the significant difference between OR and SP at the same moments: 0, 4, 8, 12, 24H); f.w., fresh weight.

Figure 2 shows the comparative evolution of the amino acid content of sourdoughs with *L. spicheri* DSM 15429 (SP) and without. *L. spicheri* DSM 15429 (OR) at different fermentation times: 0, 4, 8, 12, 24 h. 

A large body of literature showed that during sourdough fermentation with lactic acid bacteria the amino acid content improved due to the proteolytic activity of LAB [51]; Lactic acid bacteria have their own proteolytic system which may contribute to the breakdown of the proteins leading to the release of free amino acids; This complex proteolytic system is composed of a transport system, cell wall-associated proteinases, and intracellular peptidases [52]; Proteins are degraded by proteases into oligopeptides and peptidases can degrade oligopeptides into amino-acids and shorter peptides [53].

For example, *Lactobacillus sanfranciscensis* owns a proteolytic system that is able to synthesize eight amino acids, according to Papadimitrioiu et al. [54]. *Lactobacillus plantarum* CRL 778 was able to enhance the aminoacids content during fermentation of quinoa slurry [52] due to its proteolytic system and *Lactobacillus Plantarum* ATCC 8014 was able to develop and increase amino acid content during sourdough fermentation [27]. Manhoud et al. [55] showed that different strains from *Lactobacillus* and *Pediococcus* genus were able to increase the amino acid content of different cereals sourdoughs mainly due to their proteolytic activity.

Even more, according to several types of research, during the fermentation of different vegetable raw matrices with LAB, the amino acid content increase. For instance, Coda et al. [56] showed that *Lactobacillus plantarum C48* and *Lactococcus lactis* subsp. *lactis* were able to grow in the vegetable matrix such as chickpea, amaranth, quinoa, and buckwheat flours, enhancing the concentration of free amino acids and γ-aminobutyric acid. Gänzle et al. [57] reported that LAB are involved in protein degradation and amino acid metabolism due to their intracellular peptidases, increasing the concentration of amino acids.

The LAB ability to increase GABA content during fermentation depends on both the type of strain used and the matrix of the raw material. For example, the use of wholemeal flour could lead to a higher content of GABA because of the higher bran content in glutamic acid and glutamate decarboxylase [58]. Glutamic acid plays a key role in LAB aminoacids’ metabolism since almost all the aminotransferases could utilize it as a donor substrate of amino groups. LAB such as *L. plantarum* C48 and *Lactococcus lactis subsp. lactis* PU1 were able to increase the GABA content through fermentation due to glutamate decarboxylase enzyme which catalyzes the conversion of L-glutamate into GABA, [59].

In the present study, the GABA SP amount at the beginning of the fermentation was 12.93 mg/100 g product reaching a final value of 18.20 mg/100 g product after 24 h of fermentation. In the non-inoculated rice sourdough, the GABA amount increased during 24 h fermentation, reaching a value of 9.08 mg/100 g product. This could be explained by the flour endogenous glutamate decarboxylase activity [56].

The release of the phenylalanine and leucine during sourdough fermentation is increased by low pH levels [51]. The pH value of 4.1 after 24 h of fermentation allowed increasing the amount of phenylalanine and leucine (0.54 mg/100 g and 0.79 mg/100 g), reaching final values of 2.2 mg/100 g product and 1.1 mg/100 g product, respectively.

According to Zannini et al., (2012) [60], LAB can metabolize all amino acids, but the ability to degrade them varies among species. The aromatic amino acids could be degraded by LAB through transamination or decarboxylation. For example, tryptophan, tyrosine, and phenylalanine could transform indole to pyruvate, phenylpyruvate, and p-Hydroxyphenyl pyruvate through transamination reaction, catalyzed by the aromatic aminotransferase. At the same time, some strains of *Lactobacillus* could metabolize tryptophan by decarboxylation, possibly into tryptamine.

#### 3.2.2. Mineral Content of SP and OR Sourdoughs

During SP 24 h fermentation, minerals reached extended values as follows: calcium 2.3-fold higher, 1.94-fold higher for magnesium, potassium 1.98-fold higher, copper 2.55-fold higher, zinc 1.92, and manganese 1.68, as showed in Table 3.

The minerals values from OR sourdough increased 0.7–1.52-fold after 24 h of fermentation, compared with the initial fermentation time. As already proved in our study [18], during 24 h of SP fermentation the pH sourdough reached an optimum value of 4.1, compared with 5.1, reached in the case of the spontaneous fermentation (OR). The drop in the pH value showed the good adaptability of Lsp strain in the rice sourdough and allowed the enzymatic degradation of phytate, an antinutrient that is involved in the bioavailability of minerals such as iron, zinc, calcium, magnesium, potassium. Phytic acid, also known as myo-inositol 1,2,3,4,5,6-Hexakisphosphate, acts as a strong chelator of cations, being negatively charged [14].

Blandino et al., (2003) [61] reported that a reduction in phytate due to the drop in the pH value could increase several folds the number of minerals. The decreased content of phytate during sourdough fermentation thanks to the drop of the pH was reported also by Gobbetti et al., (2018) [62], Carrizo et al., (2016) [63] and Kumari et al., (2020) [64]. According to Montemurro et al., (2020) [65], who supported the idea that during LAB fermentation, the phytate complex is degraded due to the activation of endogenous and microbial phytates. Noubariene et al., (2015) [66] reported that during fermentation with various strains of *Lactobacillus* the phytate content was tremendously degraded, by 80–90%. Sourdough fermentation with *Lactobacillus panis* or *Lactobacillus* *fermentum* influences the pH decrease of the raw matrix and the phytate degradation with 90% and 70%, respectively [67].

#### 3.2.3. Total Phenolic Content and Antioxidant Activity of OR and SP Sourdough

**Figure 3:**
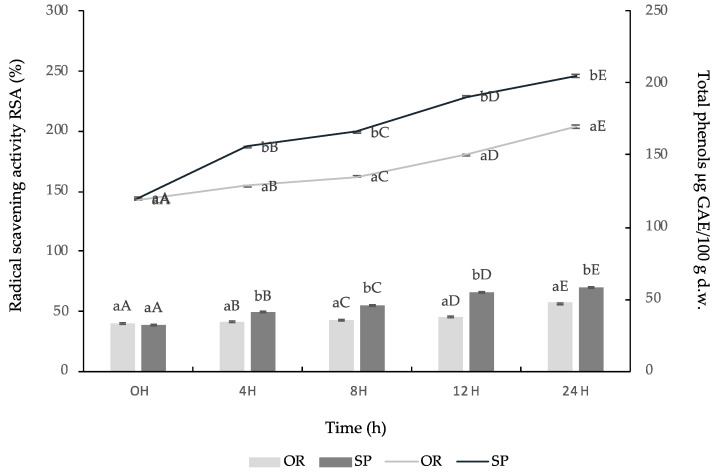
Phenolic compounds content of methanolic extracts during 0, 4, 8, 12, 24H of fermentation in inoculated (SP) and non-inoculated (OR) sourdoughs with *L. spicheri* DSM 15429, (Values not sharing the same small letters indicate the significant difference between OR and SP at different moments: 0, 4, 8, 12, 24H; Values not sharing the same capital letters indicate the significant difference between OR and SP at the same moments: 0, 4, 8, 12, 24H); d.w., dry weight.

During fermentation with the Lsp strain, an increase in the content of total phenols (TF) was observed and expressed as μg GAE/100 g d.w. (dry weight) and of radical scavenging activity (RSA%). At the beginning of the fermentation, the TF content of SP sourdough was 120.23 μg GAE/100 g d.w. and RSA was 40.30%. After 24 h of fermentation, SP reached a value of 205.03 μg GAE/100 g d.w. and 70% RSA, respectively, is significantly different from those obtained through spontaneous fermentation, having a value of 170.50 μg GAE/100 g d.w. and 57% RSA (Figure 3). These results may indicate that in the microbial activity and the endogenous enzymes of the rice flour could not significantly influence the enhancement of the antioxidant activity.

The content of phenolic acids have been several times positively correlated with the antioxidant activity as showed by Rizzello et al., (2010) [68], Montemurro et al., (2019), [64], Qui et al., (2010) [69], and Antogni et al., (2019) [70].

Other findings previously published indicate the understanding that LAB could produce the biotransformation of polyphenols into compounds with increased bioavailability and bioactivity [71].

During fermentation with lactic acid bacteria, due to the drop in the pH, the solubility and concentration of phenolic compounds increased and therefore enhanced the antioxidant activity [62]. Gobbetti et al., (2018) [72] showed that fermentation of quinoa flour with different lactic acid bacteria strains such as *Lactobacillus plantarum* T0A10, T1B6, and T6B4 enhanced the antioxidant activity of the sourdoughs.

### 3.3. Amino-Acids, Mineral, Total Phenols, Radical Scavenging Activity, and Lactic Acid Content of Final Baked Muffins

#### 3.3.1. Amino-Acids Content of Final Baked Muffins

**Figure 4:**
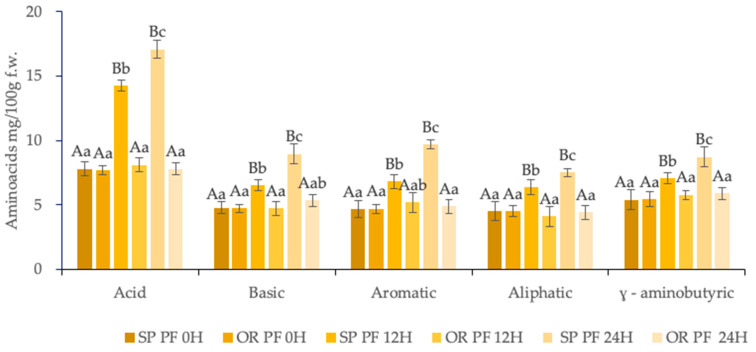
Amino acids content of final baked goods made with sourdoughs with L. spicheri DSM 15429 (SP PF) and without L. spicheri DSM 15429 (OR PF) at different fermentation times: 0, 12, 24H, (Values not sharing the same small letters indicate the significant difference between OR PF and SP PF at different moments: 0, 12, 24H; Values not sharing the same capital letters indicate the significant difference between OR and SP at the same moments: 0, 12, 24H).

In the final baked muffins SPPF 24H, as showed in Figure 4, it was observed an increment of the groups of the amino acids compared with ORPF 24H, as follows: the basic group increased 1.87-fold, the acid group with 2.28 fold, the γ-aminobutyric group with 1.61, an aliphatic group with 1.65, and aromatic with 2.07 times, as assayed in Figure 4.

The researchers’ attention was gained by γ-aminobutyric acid (GABA) due to its influence on the central nervous system acting as a major inhibitory neurotransmitters. GABA has a positive influence on human health such as depression regulation, stress, autonomic disorders [50], and may also prevent diabetes [73].

A large body of literature reported that LAB can increase GABA content of the final baked products, naturally. For instance, *Lactococcus lactis* subsp. *Lactis* was able to increase the GABA amount in fried sourdough bread (bhatura, an Indian traditional fermented food) [74]. *L. farciminis* and *L. brevis species* were also able to increase GABA content during sourdough fermentation and improved the GABA content of the final baked goods [75]. The ability of *Lactobacillus plantarum* ATCC 8014 to increase GABA content in quinoa sourdough and the final baked muffins was also shown in our previous study [27]. In the present study, GABA content of the final baked product SP PF 24H increased up to 3.96 mg/ 100 product, being statistically different (*p* < 0.05) from OR PF 24H with a final value of 2.53 mg/100 g product.

Proteolysis activity of LAB plays paramount roles in the formation of the aroma of the final product, mainly because of the amino acids generated during fermentation which act as a major precursor of specific flavor compounds [76]. Ashaolu et al., (2020) [77] supported the idea that cereal fermentation with different LAB could be a suitable form to improve the flavor of the final baked goods, due to the accumulation of amino-acids during fermentation which could be considered as flavor precursor compounds or taste-active compounds. Isoleucine, leucine, and valine are precursors amino acids of volatile aroma compounds such as acids, aldehydes, alcohols, and esters and could influence the aroma of the final baked goods [60]. Adding SP 24H sourdough increased in the final baked product the content of isoleucine, leucine, and valine up to 0.31 g/100 g protein, 0.72 g/100 g protein and 0.21 g/100 g protein, respectively.

Even more, fermentation enhances the taste of the final products, increases the essential free amino acid content, total acids, and being involved in the nutritional profile of the products [60]. Due to the ability of LAB to metabolize different functional components of foods the formation of free amino acids could be explained [66].

Coda et al., (2010) [5] showed that different strains of lactic acid bacteria could enhance the amino acid content, on vegetable matrices such as chickpea, lentil, and bean flours, improving the nutritional, texture, and sensory characteristics of final baked goods. In our previous work, Chiș et al., (2020) [18], we already showed that the rice sourdough fermented with *Lactobacillus spicheri* DMS 1549 was able to improve textural and sensorial properties of gluten-free baked muffins through the influence on the hardness, cohesiveness, springiness, and resilience and due to the pleasant aroma compounds formed during fermentation and baking. The hedonic test was used to establish hedonic scores for muffins’ sensory characteristics such as appearance, color, texture, taste, flavor, texture, and overall acceptability. The muffins sample SP PF 24H was the most appreciated by the consumers (hedonic score 8.3).

#### 3.3.2. Minerals Final Baked Muffins Content

The minerals content of the SP PF 12H and SP PF 24H were statistically different (*p* < 0.05) from OR PF 12H and ORPF 24H, respectively, as reported in Table 4. Mg and K were found in the biggest amounts, followed by Ca, Fe, Zn, Mn, and Cu. Minerals such as Ca and Mg are playing an important role in human metabolism, influencing the bone structure and the water and salt balance [74], meanwhile Zn deficiency could include dry skin and brittle nails [4] and might be responsible for respiratory tract infections and diarrheal disease [28]. In some countries, iron is used for the fortification of wheat and corn flours to prevent the anemia occurrence [78].

The results of the mineral content obtained in the present study agree with the report of Abosede et al., (2019) [79] who supported that the use of sourdough fermented with LAB strains is an important source of minerals and enriched the final content of calcium and magnesium in the baked goods.

The influence of the raw matrix on the final mineral content of the final baked goods was shown also by Lopez et al., (2013) [80] and Gobbetti et al., (2013) [73] highlighting that sourdough could play a key role in the minerals content of the products.

#### 3.3.3. Lactic Acid Content

In our previous findings Chiș et al., (2020) [18], we proved that after 24 h of the rice sourdough fermentation with Lsp there was an augmentation of the lactic acid content up to 15 mmol/L, in comparison to spontaneous fermentation (3.1 mmol/L lactic acid). This could be explained by the capacity of Lsp strain to ferment the rice carbon sources to produce lactic acid which leads to the decrease of the pH.

**Figure 5:**
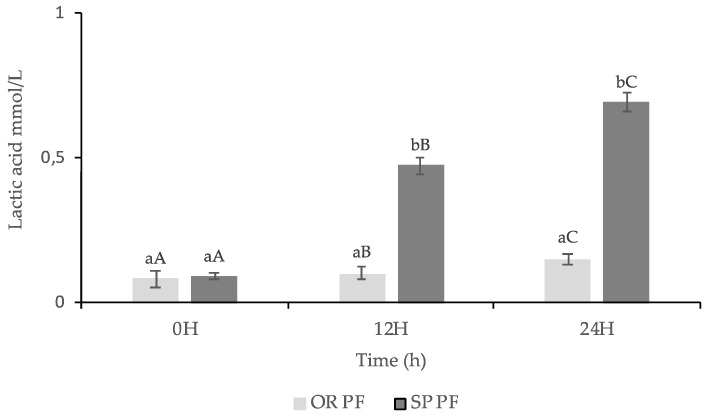
The lactic acid content of final baked goods made with sourdoughs with *L. spicheri* DSM 15429 (SP PF) and without *L. spicheri* DSM 15429 (OR PF) at different fermentation times: 0, 12, 24H, (Values not sharing the same small letters indicate the significant difference between OR PF and SPPF at the same moment: 0, 12, 24H; Values not sharing the same capital letters indicate the significant difference between OR PF and SPPF at different moments: 0, 12, 24H).

The lactic acid in the two final baked products presented in Figure 5 were statistically different: 0.69 mmol/L for SPPF 24 h and 0.15 mmol/L for OR PF 24H. The Pearson correlation value of 0.96 showed a strong relationship between the lactic acid content of the final baked goods and the content of SP sourdough.

Other published data confirm that lactic acid content of the final baked goods depends on the lactic acid sourdough content [54,62,81] improving the safety and the storage quality of it [50].

The presence of lactic acid in final baked goods could influence the preservation of the final baked goods through the inhibition of yeast, molds, and Gram-positive and Gram-negative bacteria development [82,83,84], and could reduce acute glycemic and/or insulinemic responses [59].

As showed in Table 5, during 9 storage days of the final baked goods, the total viable count of yeasts and molds (TYMC) in the case of SP PF products was significantly different (*p* < 0.05) from the OR PF content. This could be due to the presence of lactic acid in the sourdough with an influence on the final baked lactic acid amount.

#### 3.3.4. Total Phenolic Content and Antioxidant Activity of Final Baked Goods

Rizzello et al., (2016) [85] showed that *Lactobacillus plantarum* T6B10 and *Lactobacillus rossiae* T0A16 were able to grow in raw matrix, enhancing the antioxidant activity and total phenols content of the final baked good. By the lactic acid pathway, LAB could influence the solubilization of the phenolic compounds. Even more, the use of quinoa sourdough fermented with *Lb. plantarum* strains in the manufacture of the white bread improved the nutritional (free amino acids, antioxidant activity, organic acids, total phenols), textural and sensory features of the final baked good [85].

**Figure 6:**
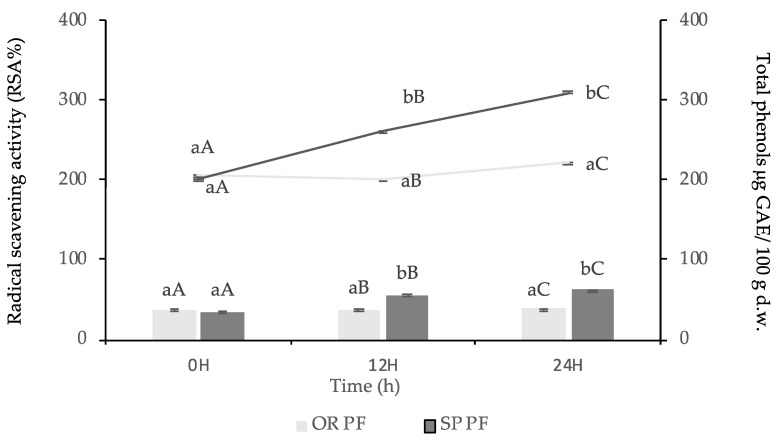
Radical scavenging activity and phenolic compounds content of final baked goods methanolic extracts during 0, 12, 24H made with sourdoughs with *L. spicheri* DSM 15429 (SP PF) and without *L. spicheri* DSM 15429 (OR PF), (Values not sharing the same small letters indicate the significant difference between OR and SP at the same moment: 0, 12, 24H, (Values not sharing the same capital letters indicate the difference between OR and SP at different moments: 0, 12, 24H); d.w., dry weight.

In the present study, the RSA and TF amounts of the final baked goods OR PF 12H, OR PF 24H and SP PF 12H and SP PF 24H, respectively, were statistically significantly different (*p* < 0.05), as shown in Figure 6. The RSA and TF from SP PF 24H was 62% and 310.48 mg GAE/100 g d.w., compared with OR PF 24 H, were the values were 40% and 221.04 mg GAE/100 g d.w., respectively. High Pearson’s values (0.96, 0.90) indicated a strong relationship between the increased amounts of RSA and TF manufactured with SP sourdough at different times: 0, 12, 24 h. Usually, the increasing amount of total phenols and antioxidant activity in final baked goods is due to the sourdough fermentation with lactic acid bacteria [68]. Even more, according to Samadi et al., (2010) [86], amino-acids such as methionine, lysine, tryptophan, and histidine can enhance antioxidant activity being able to donate protons to electron-deficient radicals. These amino-acids could act as inhibitors of lipid peroxidation and scavengers of free radicals. Even if many researchers have paid close attention to this mechanism until now it is not fully comprehended [87].

From the technological point of view, antioxidants act as food preservatives in many ways: inhibit oxidation and food spoilage, as well as maintain taste, aroma, and freshness of the final baked good [87].

## 4. Conclusions

An abundant literature emphasizes the importance of LAB fermentation on the nutritional features of the final baked goods. Even if the sourdough fermentation is fully studied, it still needs a thorough exploitation. In the present research, the effect of rice flour fermentation with Lsp strain and of spontaneous fermentation on the nutritional characteristics of the final baked goods was studied. The addition of 15% rice fermented sourdough with Lsp strain was successful in improving the nutritional characteristics of the gluten-free muffins. The nutritional profile of the rice flour fermented with the Lsp strain was improved. Gluten-free muffins manufactured with rice flour fermented with Lsp strain presented a higher nutritional profile compared with the rice flour spontaneous fermentation. Amino acids, minerals, lactic acid, total phenols, and antioxidant potential contents enhanced their values, showing that the Lsp strain could be used to improve the nutritional values of gluten-free muffins. From the RWF 21 amino-acids identified and grouped in acid, basic, aromatic, aliphatic, and γ-aminobutyric acid, the most representative were aspartic, arginine, tyrosine, alanine, and γ-aminobutyric acid. During the fermentation with the Lsp strain, the amino-acid content increased, possibly due to the proteolytic system of this strain. From the RWF minerals content, K, Mg, and Ca were the most representative and increased their value during SP fermentation, due to the drop of the pH which leads to the possible degradation of phytic acid. Furthermore, the presence of lactic acid in the final baked product had a positive influence on the shelf life of the final baked muffins.

These findings may contribute to the development of a new range of gluten-free products based on the fermentation of rice flour with different LAB strains, aiming to improve their nutritional values.

## Figures and Tables

**Table 1:** Amino acid content of rice wholemeal flour (RWF).

Amino Acids		RWF (mg/100 g f.w.)
Acid group	Glutamine	14.11 ± 0.12
	Asparagine	25.53 ± 0.31
	Glutamic acid	32.22 ± 0.11
	Aspartic acid	37.05 ± 0.21
Basic group	Histidine	1.92 ± 0.10
	Lysine	5.50 ± 0.13
	Ornithine	0.74 ± 0.09
	Arginine	11.12 ± 0.13
Aromatic group	Tryptophan	1.74 ± 0.17
	Tyrosine	2.63 ± 0.14
	Phenylalanine	1.50 ± 0.21
Aliphatic group	Prolamine	3.10 ± 0.13
	Isoleucine	0.88 ± 0.07
	Leucine	1.84 ± 0.05
	Valine	1.95 ± 0.03
	Alanine	7.61 ± 0.04
	Glycine	2.28 ± 0.02
γ-aminobutyric group	Proline	3.10 ± 0.03
	Threonine	2.61 ± 0.02
	Serine	6.20 ± 0.02
	γ-aminobutyric acid	12.55 ± 0.5
∑Acid group		108.91 ± 0.75
∑Basic group		19.38 ± 0.45
∑Aromatic group		5.87 ± 0.52
∑Aliphatic group		17.66 ± 0.34
∑γ-aminobutyric group		24.46 ± 0.87

Mean values of three different determinations followed by standard deviation; f.w., fresh weight.

**Table 2:** Chemical composition and mineral content of rice wholemeal flour (RWF).

Parameters	RWF
Minerals, mg/100 g f.w.	
Ca	15.02 ± 0.20
Mg	231.36 ± 0.47
K	437.18 ± 0.26
Fe	1.21 ± 0.14
Cu	0.35 ± 0.01
Zn	0.97 ± 0.04
Mn	1.14 ±0.02
Cr	0.016 ± 0.01

Mean values of three different determinations followed by standard deviation; f.w., fresh weight.

**Table 3:** Minerals (mg/100 g f.w.) amount during OR and SP fermentation *.

Samples	Ca	Mg	K	Fe	Cu	Zn	Mn
OR 0H	7.92 ± 0.04 ^A^^a^	50.00 ± 0.23 ^Aa^	200.32 ± 0.56 ^A^^a^	0.42 ± 0.03 ^A^^a^	0.09 ± 0.02 ^A^^bc^	0.39 ± 0.02 ^A^^a^	0.79 ± 0.03 ^A^^d^
SP 0H	7.90 ± 0.31 ^A^^a^	50.01 ± 0.12 ^A^^a^	199.32 ± 0.19 ^A^^a^	0.42 ± 0.02 ^A^^a^	0.08 ± 0.01 ^A^^a^	0.39 ± 0.01 ^A^^a^	0.79 ± 0.02 ^A^^d^
OR 4H	8.21 ± 0.23 ^A^^ab^	55.80 ± 0.32 ^A^^b^	205.50 ± 0.45 ^A^^a^	0.44 ± 0.01 ^A^^ab^	0.12 ± 0.01 ^A^^bc^	0.43 ± 0.02 ^A^^bc^	0.29 ± 0.02 ^A^^b^
SP 4H	8.40 ± 0.12 ^A^^abc^	70.00 ± 0.25 ^B^^c^	220.31 ± 0.37 ^B^^bc^	0.45 ± 0.11 ^A^^ab^	0.09 ± 0.03 ^A^^ab^	0.42 ± 0.02 ^A^^b^	0.80 ± 0.03 ^Bd^
OR 8H	8.50 ± 0.11 ^A^^abc^	57.31± 0.67 ^A^^b^	210.10 ± 0.45 ^A^^ab^	0.45 ± 0.10 ^A^^ab^	0.11 ± 0.02 ^A^^cd^	0.45 ± 0.03 ^A^^c^	0.35 ± 0.04 ^A^^b^
SP 8H	9.01 ± 0.54 ^A^^bc^	80.00 ± 0.89 ^B^^d^	240.90 ± 0.78 ^B^^d^	0.56 ± 0.03 ^A^^c^	0.13 ± 0.01 ^A^^ef^	0.49 ± 0.03 ^B^^d^	0.81 ± 0.06 ^B^^d^
OR 12H	8.92 ± 0.32 ^A^^bc^	60.91 ± 0.45 ^A^^c^	226.80 ± 0.38 ^A^^c^	0.47 ± 0.03 ^A^^b^	0.12^d^ ± 0.03 ^A^^e^	0.47 ± 0.02 ^Ad^	0.59 ± 0.03 ^A^^c^
SP 12H	9.31 ± 0.51 ^B^^c^	90.48 ± 0.37 ^B^^e^	299.63 ± 0.59 ^B^^f^	0.76 ± 0.06 ^B^^e^	0.17 ± 0.01 ^B^^g^	0.62 ± 0.01 ^B^^f^	0.81 ± 0.07 ^Bd^
OR 24H	9.15 ± 0.25 ^A^^c^	68.32 ± 0.52 ^A^^d^	255.95 ± 0.16 ^A^^e^	0.64 ± 0.01 ^A^^d^	0.13 ± 0.01 ^A^^f^	0.51 ± 0.02 ^A^^e^	1.16 ± 0.07 ^A^^e^
SP 24H	18.19 ± 0.51 ^B^^d^	97.49 ± 0.71 ^B^^f^	395.11 ± 0.32 ^B^^g^	0.96 ± 0.03 ^B^^f^	0.25 ± 0.02 ^B^^h^	0.75 ± 0.04 ^B^^g^	1.33 ± 0.08 ^Bf^

*, different small letters (a–f) indicate the significant difference for the same compounds between OR and SP minerals at different moments: 0, 4, 8, 12, 24H; different capital letters (A–B) indicate the significant difference QP for the same compounds between OR and QSP minerals at the same moment: 0, 4, 8, 12, 24H; f.w., fresh weigh; each value was the mean of duplicate measurements.

**Table 4:** Mineral content (mg/100 g f.w.) of ORPF and SPPF muffins *.

Samples	K	Mg	Ca	Mn	Fe	Zn	Cu
OR SP 0H	389.23 ± 1.20 ^Aa^	142.56 ± 0.67 ^Aa^	11.32 ± 0.56 ^Aa^	0.85 ± 0.3 ^Aa^	0.91 ± 0.32 ^Aa^	0.85 ± 0.31 ^Aa^	0.34 ± 0.01 ^Aa^
SP PF 0H	390.23 ± 1.01 ^Aa^	140.32 ± 0.99 ^Aa^	11.32a ± 0.34 ^Aa^	0.87 ± 0.01 ^Aa^	0.90 ± 0.02 ^Aa^	0.84 ± 0.03^Aa^	0.35 ± 0.01 ^Aa^
OR PF 12H	400.66 ± 0.67 ^Aa^	158.84 ± 1.20 ^Ab^	12.02 ± 0.12 ^Aa^	0.91 ± 0.05 ^Aa^	0.98 ± 0.22 ^Aab^	0.90 ± 0.01 ^Aab^	0.36 ± 0.01 ^Aa^
SP PF 12H	437.70 ± 0.55 ^Bb^	175.70 ± 0.34 ^Bc^	14.66 ± 0.22 ^Bbc^	1.14 ± 0.04 ^Bb^	1.20 ± 0.21 ^Bb^	1.19 ± 0.03 ^Bc^	0.44 ± 0.01 ^Ab^
OR PF 24H	410.10 ± 0.34 ^Aab^	177.81 ± 0.32 ^Ac^	13.06a ± 0.35 ^Ab^	0.95 ± 0.01 ^Aa^	1.01 ± 0.02 ^Aab^	0.95 ± 0.04^Aab^	0.39 ± 0.02^Aab^
SP PF 24H	481.11 ± 0.12 ^Bc^	190.99 ± 0.22 ^Bd^	16.27 ± 1.01 ^Bc^	1.35 ± 0.04 ^Bc^	1.49 ± 0.03 ^Bc^	1.47 ± 0.02 ^Bd^	0.53 ± 0.04 ^Bc^

*, different small letters (a–d) indicate the significant difference between ORPF and SPPF minerals at different moments: 0, 12, 24H, (Values not sharing the same capital letters (A–B) indicate the significant difference between OR and SP at the same moments: 0, 12, 24H); Each value was the mean of duplicate measurements; f.w., fresh weight.

**Table 5:** Total count of yeasts and molds (TYMC) evolution (cfu/g) during storage days of the final products *.

Storage (Days)	Parameter	OR PF 0H	SP PF 0H	OR PF 12H	SP PF 12H	OR PF 24H	SP PF 24H
Day 1	TYMC, cfu/g	5.6 ± 0.02 ^Aa^	5.7 ± 0.25 ^Aa^	7.4 ± 0.49 ^Bc^	6.7 ± 0.49 ^Aab^	12.5 ± 0.56 ^Ae^	10.4 ± 0.64^Ad^
Day 3	TYMC, cfu/g	15.6 ± 0.04 ^Aa^	15.4 ± 0.89 ^Aa^	14.7 ± 0.78 ^Bc^	12.9 ± 0.55 ^Ab^	15.3 ± 0.55 ^Bbc^	12.6 ± 0.67^Ab^
Day 5	TYMC, cfu/g	40.3 ± 0.12 ^Ae^	40.5 ± 0.56 ^Ae^	38 ± 0.89 ^Bd^	28.57 ± 0.89 ^Aa^	35.7 ± 0.69 ^Bc^	30 ± 0.89 ^Aab^
Day 7	TYMC, cfu/g	60.3 ± 1.00 ^Ae^	60.5 ± 0.28 ^Ae^	45 ± 0.95 ^Bc^	32 ± 0.77 ^Aa^	50.9 ± 0.42^Bd^	40 ± 1.00 ^Ab^
Day 9	TYMC, cfu/g	110.3 ± 0.98 ^Bef^	109 ± 0.45 ^Ae^	90.8 ± 1.5 ^Bc^	70.6 ± 1.2 ^Ab^	100.8 ± 1.4 ^Bd^	60.6 ± 0.99 ^Aa^

*, different small letters (a–f) indicate the significant difference between ORPF and SPPF TYMC evolution at different moments: 0, 12, 24H, (Values not sharing the same capital letters (A–B)indicate the significant difference between ORPF and SPPF TYMC at the same moments: 0, 12, 24H); Each value was the mean of duplicate measurements.

## References

[1] Codex Alimentarius CODEX STAN 118-1979: Standard for Foods for Special Dietary Use for Persons Intolerant to Gluten. http://www.fao.org/fao-who-codexalimentarius/standards/en/.

[2] Aponte M., Boscaino F., Sorrentino A., Coppola R., Masi P., Romano A. (2013). Volatile compounds and bacterial community dynamics of chestnut-flour-based sourdoughs. Food Chem..

[3] Ziobro R., Juszczak L., Witczak M., Korus J. (2015). Non-gluten proteins as structure forming agents in gluten free bread. J. Food Sci. Technol..

[4] Theethira T.G., Dennis M., Leffler D.A., Daniel A. (2014). Nutritional consequences of celiac disease and the gluten-free diet. Expert Rev. Gastroenterol. Hepatol..

[5] Cao X., Wen H., Li C., Gu Z. (2009). Differences in functional properties and biochemical characteristics of congenetic rice proteins. J. Cereal Sci..

[6] Singh A., Matta N. (2011). Disulphide linkages occur in many polypeptides of rice protein fractions: A two-dimensional gel electrophoretic study. Rice Sci..

[7] Chan Y.J., Lu W.C., Lin H.Y., Wu Z.R., Liou C.W., Li P.H. (2020). Effect of Rice Protein Hydrolysates as an Egg Replacement on the Physicochemical Properties of Flaky Egg Rolls. Foods.

[8] Ilowefah M., Chinma C., Bakar J., Hasanah M., Ghazali H.M., Muhammad K., Makeri M. (2014). Fermented Brown Rice Flour as Functional Food Ingredient. Foods.

[9] Chan K.W., Khong N.M.H., Iqbal S., Ismail M. (2012). Simulated Gastrointestinal pH Condition Improves Antioxidant Properties of Wheat and Rice Flours. Int. J. Mol. Sci..

[10] Gujral H.S., Guardiola I., Carbonell J.V., Rosell C.M. (2003). Effect of cyclodextrinase on dough rheology and bread quality from rice flour. J. Agric. Food Chem..

[11] Mert S., Sahin S., Sumnu G. (2015). Development of Gluten-Free Wafer Sheet Formulations. Food Sci. Technol..

[12] Monk J.L.M., Vanier N.L., Casaril J., Berto R.M., De Oliveira M., Gomes C.B., De Carvalho M.P., Guerra Dias A.R., Elias M.C. (2013). Effects of milling on proximate composition, folic acid, fatty acids and technological properties of rice. J. Food Compos. Anal..

[13] Păucean A., Man S., Muste S., Pop A. (2015). Effect of quinoa flour addition on quality characteristics of rice gluten-free cookies. J. Agroaliment. Process. Technol..

[14] Perera I., Seneweera S., Hirotsu N. (2018). Manipulating the Phytic Acid Content of Rice Grain Toward Improving Micronutrient Bioavailability. Rice.

[15] Vogelmann S.A., Seitter M., Singer U., Brandt M.J., Hertel C. (2009). Adaptability of lactic acid bacteria and yeasts to sourdoughs prepared from cereals, pseudocereals and cassava and use of competitive strains as starters. Int. J. Food Microbiol..

[16] Coda R., Di Cagno R., Gobbetti M., Rizzello C.G. (2014). Sourdough lactic acid bacteria: Exploration of non-wheat cereal-based fermentation. Food Microbiol..

[17] Lee S.M., Hwang Y.R., Kim M.S., Chung M.S., Kim Y.S. (2019). Comparison of Volatile and Nonvolatile Compounds in Rice Fermented by Different Lactic Acid Bacteria. Molecules.

[18] Chis M.S., Păucean A., Man S.M., Muresan V., Socaci S.A., Pop A., Stan L., Rusu B., Muste S. (2020). Textural and Sensory Features Changes of Gluten Free Muffins Based on Rice Sourdough Fermented with *Lactobacillus spicheri* DSM 15429. Foods.

[19] Balli D., Bellumori M., Paoli P., Pieraccini G., Di Paola M., De Filippo C., Di Gioia D., Mulinacci N., Innocenti M. (2019). Study on a Fermented Whole Wheat: Phenolic Content, Activity on PTP1B Enzyme and In Vitro Prebiotic Properties. Molecules.

[20] Ilowefah M., Bakar J., Ghazali H.M., Mediani A., Muhammad K. (2014). Physicochemical and functional properties of yeast fermented brown rice flour. J. Food Sci. Technol..

[21] Teleky B.E., Martău A.G., Ranga F., Chețan F., Vodnar D.C. (2020). Exploitation of Lactic Acid Bacteria and Baker’s Yeast as Single or Multiple Starter Cultures of Wheat Flour Dough Enriched with Soy Flour. Biomolecules.

[22] Biscola V., Albuquerque M.A.C., Nunes T.P., Vieira A.D.S., Franco D.G.M., Albuquerque M.A.C., LeBlanc A.M., LeBlanc J.G., Bedani R. (2020). Lactic Acid Bacteria: A Functional Approach.

[23] Maidana D.S., Finch S., Garro M., Savoy G., Ganzle M., Vignolo G. (2020). Development of gluten-free breads started with chia and flaxseed sourdoughs fermented by selected lactic acid bacteria. LWT Food Sci. Technol..

[24] Meroth C.B., Hammes W.P., Hertel C. (2004). Characterisation of the microbiota of rice sourdoughs and description of *Lactobacillus spicheri* sp. nov. Syst. Appl. Microbiol..

[25] Bourekoua H., Benatallah L., Zidoune M.N., Rosell C.M. (2016). Developing gluten free bakery improvers by hydrothermal treatment of rice and corn flours. Lwt Food Sci. Technol..

[26] Bobis O., Dezmirean D.S., Mărghitaș L.A., Bonta V., Urcan A., Pașca C., Moise A.R. (2018). *Morus* sp. for revigorating silkworm breeding in Romania and promoting health benefits of leaves and fruits. Sci. Papers. Series B Hortic..

[27] Chiş M.S., Păucean A., Stan L., Suharoschi R., Man S.M., Muste S. (2018). Protein metabolic conversion of nutritional features during quinoa sourdough fermentation and its impact on baked goods. Cyta J. Food.

[28] Păucean A., Moldovan O.P., Mureşan V., Socaci S.A., Dulf F., Man M.S., Mureşan A.E., Muste S. (2018). Folic acid, minerals, amino-acids, fatty acids and volatile compounds of green and red lentils. Folic acid content optimization in wheat-lentils composite flours. Chem. Cent. J..

[29] Mihoc M., Pop G., Alexa E., Radulov I. (2012). Nutritive quality of romanian hemp varieties (*Cannabis sativa L.*) with special focus on oil and metal contents of seeds. Chem. Cent. J..

[30] Păucean A., Vodnar D.C., Socaci S.A., Socaciu C. (2013). Carbohydrate metabolic conversions to lactic acid and volatile derivatives, as influenced by *Lactobacillus plantarum* ATCC 8014 and *Lactobacillus casei* ATCC 393 efficiency during in vitro and sourdough fermentation. Eur. Food Res. Technol..

[31] Bunea A., Ruginǎ D.O., Pintea A.M., Sconţa Z., Bunea C.I., Socaciu C. (2011). Comparative polyphenolic content and antioxidant activities of some wild and cultivated blueberries from Romania. Not. Bot. Horti Agrobot..

[32] Păucean A., Man S.M., Chis M.S., Mureşan V., Pop C.R., Socaci S.A., Mureşan C.C., Muste S. (2019). Use of pseudocereals preferment made with aromatic yeast strains for enhancing wheat bread quality. Foods.

[33] Dordević T.M., Šiler-Marinković S.S., Dimitrijević-Branković S.I. (2010). Effect of fermentation on antioxidant properties of some cereals and pseudo cereals. Food Chem..

[34] Chiş M.S., Păucean A., Stan L., Mureşan V., Vlaic R.A., Man S., Biriş-Dorhoi S.E., Muste S. (2018). *Lactobacillus plantarum* ATCC 8014 in quinoa sourdough adaptability and antioxidant potential. Rom. Biotech. Lett..

[35] International Organization for Standardization (2008). International Standard ISO 21527-2.

[36] Antoniewska A., Rutkowska J., Pineda M.M., Adamska A. (2018). Antioxidative, nutritional and sensory properties of muffins with buckwheat flakes and amaranth flour blend partially substituting for wheat flour. Lwt Food Sci. Technol..

[37] Nagy M., Semeniuc C.A., Socaci A.M., Pop C.R., Rotar A.M., Sălagean C.D., Tofană M. (2017). Utilization of brewer’s spent grain and mushrooms in fortification of smoked sausages. Food Sci. Technol..

[38] Kati K., Kaisa P., Karin A. (2004). Influence and Interactions of Processing Conditions and Starter Culture on Formation of Acids, Volatile Compounds, and Amino Acids in Wheat Sourdoughs. Cereal Chem..

[39] Amagliani L., O’Regan J., Kelly A.L., O’Mahony J.A. (2017). Composition and protein profile analysis of rice protein ingredients. J. Food Compos. Anal..

[40] Liang J., Han B., Han L., Nout M.J.R., Hamer R.J. (2007). Iron, zinc and phytic acid content of selected rice varieties from China. J. Sci. Food Agric..

[41] Oko A.O., Ubi B.E., Efisue A.A., Dambaba N. (2012). Comparative Analysis of the Chemical Nutrient Composition of Selected Local and Newly Introduced Rice Varieties Grown in Ebonyi State of Nigeria. Int. J. Agric. For..

[42] Verma D.K., Srivastav P.P. (2017). Proximate Composition, Mineral Content and Fatty Acids Analyses of Aromatic and Non-Aromatic Indian Rice. Rice Sci..

[43] Mbatchou V.C., Dawda S. (2013). The Nutritional Composition of Four Rice Varieties Grown and Used in Different Food Preparations in Kassena-Nankana District, Ghana. Int. J. Res. Chem. Environ..

[44] Heinemann R.J.B., Fagundes P.L., Pinto E.A., Penteado M.V.C. (2015). Comparative study of nutrient composition of commercial brown, parboiled and milled rice from Brazil. J. Food Compos. Anal..

[45] Pang Y., Ahmed S., Xu Y., Beta T., Zhu Z., Shao Y. (2018). Bound phenolic compounds and antioxidant properties of whole grain and bran of white, red and black rice. Food Chem..

[46] Sakač M., Pestorić M., Mišan A., Nedeljković N., Jambrec D., Jovanov P., Banjac V., Torbica A., Hadnađev M., Mandić A. (2015). Antioxidant capacity, mineral content and sensory properties of gluten-free rice and buckwheat cookies. Food Technol. Biotechnol..

[47] Butsat S., Siriamornpun S. (2010). Antioxidant capacities and phenolic compounds of the husk, bran and endosperm of Thai rice. Food Chem..

[48] Gorinstein S., Vargas O.J.M., Jaramillo N.O., Sallas I.A., Ayala A.L.M., Arancibia-Avila P., Toledo F., Katrich E., Trakhtenberg S. (2007). The total polyphenols and the antioxidant potentials of some selected cereals and pseudocereals. Eur. Food Res. Technol..

[49] Fitriani D.R., Rumpagaporn P. Antioxidant Activity of Enzymatically Treated Extracts from Commercially Defatted Rice Bran. Agricultural Sciences: Leading Thailand to World Class Standards. Proceedings of the 52nd Kasetsart University Annual Conference.

[50] Lloyd B.J., Siebenmorgen T.J., Beers K.W. (2000). Effects of commercial processing on antioxidants in rice bran. Cereal Chem..

[51] Thiele C., Gänzle M.G., Vogel R.F. (2002). Contribution of sourdough lactobacilli, yeast, and cereal enzymes to the generation of amino acids in dough relevant for bread flavor. Cereal Chem..

[52] Dallagnol A.M., Pescuma M., De Valdez G.E., Rollán G. (2013). Fermentation of quinoa and wheat slurries by *Lactobacillus plantarum* CRL 778: Proteolytic activity. Appl. Microbiol. Biotechnol..

[53] Ruiz-Rodríguez L., Bleckwedel J., Ortiz M.E., Pescuma M., Mozzi F., Wittmann C., Liao J.C. (2016). Lactic Acid Bacteria, Industrial Biotechnology: Microorganisms.

[54] Papadimitriou K., Zoumpopoulou G., Georgalaki M., Alexandraki V., Kazou M., Anastasiou R., Tsakalidou E., Galanakis C.M. (2019). Sourdough Bread, Innovations in Traditional Foods.

[55] Mamhoud A., Nionelli L., Bouzaine T., Hamdi M., Gobbetti M., Rizzello C.G. (2016). Selection of lactic acid bacteria isolated from tunisian cereals and exploitation of the use as starters for sourdough fermentation. Int. J. Food Microbiol..

[56] Coda R., Rizzello C.G., Gobbetti M. (2010). Use of sourdough fermentation and pseudo-cereals and leguminous flours for the making of a functional bread enriched of -aminobutyric acid (GABA). Int. J. Food Microbiol..

[57] Gänzle M.G. (2014). Enzymatic and bacterial conversions during sourdough fermentation. Food Microbiol..

[58] Di Cagno R., Rizzello C.G., De Angelis M., Cassone A., Giuliani G., Benedusi A., Limitone A., Surico M.F., Gobbetti M. (2008). Use of selected sourdough strains of *Lactobacillus* for removing gluten and enhancing the nutritional properties of gluten-free bread. J. Food Prot..

[59] Zannini E., Pontonio E., Waters D.M., Arendt E.K. (2012). Applications of microbial fermentations for production of gluten-free products and perspectives. Appl. Microbiol. Biotechnol..

[60] Fernández M., Zúñiga M. (2006). Amino acid catabolic pathways of lactic acid bacteria. Crit. Rev. Microbiol..

[61] Blandino A., Al-Aseeri M.E., Pandiella S.S., Cantero D., Webb C. (2003). Cereal-based fermented foods and beverages. Food Res. Int..

[62] Gobbetti M., De Angelis M., Corsetti A., Di Cagno R., Calasso R., Archetti G., Rizzello C.G. (2019). Novel insights on the functional/nutritional features of the sourdough fermentation. Int. J. Food Microbiol..

[63] Carrizo S.L., Montes de Oca C.E., Laiño J.E., Suarez N.E., Vignolo G., LeBlanc J.G., Rollán G. (2016). Ancestral Andean grain quinoa as source of lactic acid bacteria capable to degrade phytate and produce B-group vitamins. Food Res. Int..

[64] Kumari S., Bhinder S., Singh B., Kaur A., Singh N. (2020). Effect of buckwheat incorporation on batter fermentation, rheology, phenolic, amino acid composition and textural properties of idli. LWT Food Sci. Technol..

[65] Montemurro M., Coda R., Rizzello C.G. (2019). Recent Advances in the Use of SourdoughBiotechnology in Pasta Making. Foods.

[66] Sharma N., Angural S., Rana M., Puri N., Kondepudi K.K., Gupta N. (2020). Phytase producing lactic acid bacteria: Cell factories for enhancing micronutrient bioavailability of phytate rich foods. Trends Food Sci. Technol..

[67] Nuobariene L., Cizeikiene D., Gradzeviciute E., Hansen A.E., Rasmussen S.K., Juodeikiene G., Vogensen F.K. (2015). Phytase-active lactic acid bacteria from sourdoughs: Isolation and identification. LWT Food Sci. Technol..

[68] Rizzello C.G., Nionelli L., Coda R., De Angelis M., Gobbetti M. (2010). Effect of sourdough fermentation on stabilisation, and chemical and nutritional characteristics of wheat germ. Food Chem..

[69] Qiu Y., Liu Q., Beta T. (2010). Antioxidant properties of commercial wild rice and analysis of soluble and insoluble phenolic acids. Food Chem..

[70] Antognoni F., Mandrioli R., Potente G., Taneyo Saa D.L., Gianotti A. (2019). Changes in carotenoids, phenolic acids and antioxidant capacity in bread wheat doughs fermented with different lactic acid bacteria strains. Food Chem..

[71] Li Z., Teng J., Lyu Y., Hu X., Zhao Y. (2019). Enhanced Antioxidant Activity for Apple Juice. Molecules.

[72] Rizzello C.G., Lorusso A., Russo V., Pinto D., Marzani B., Gobbetti M. (2017). Improving the antioxidant properties of quinoa flour through fermentation with selected autochthonous lactic acid bacteria. Int. J. Food Microbiol..

[73] Gobbetti M., Rizzello C.G., Di Cagno R., De Angelis M. (2014). How the sourdough may affect the functional features of leavened baked goods. Food Microbiol..

[74] Bhanwar S., Bamnia M., Ghosh M., Ganguli A. (2013). Use of *Lactococcus lactis* to enrich sourdough bread with γ-aminobutyric acid. Int. J. Food Sci. Nutr..

[75] Venturi M., Galli V., Pini N., Guerrini S., Granchi L. (2019). Use of selected lactobacilli to increase γ-Aminobutyric acid (GABA) content in sourdough bread enriched with amaranth flour. Foods.

[76] Ashaolu T.J. (2020). Safety and quality of bacterially fermented functional foods and beverages: A mini review. Food Qual. Saf..

[77] Zhao C.J., Schieber A., Gänzle M.G. (2016). Formation of taste-active amino acids, amino acid derivatives and peptides in food fermentations—A review. Food. Res. Int..

[78] Rebellato A.P., Pacheco B.C., Prado J.P., Lima Pallone J.A. (2015). Iron in fortified biscuits: A simple method for its quantification, bioaccessibility study and physicochemical quality. Food Res. Int..

[79] Abosede A.M., Ifesan B.O.T., Enujiugha V.N., Adefisola B.A. (2019). Microbiological and Physicochemical Properties of Wholegrain Millet Sourdough Breads. Int. J. Food Sci. Nutr..

[80] Lopez H.W., Duclos V., Coudray C., Krespine V., Feillet-Coudray C., Messager A., Demigne’ C., Remesy C. (2013). Making bread with sourdough improves mineral bioavailability from reconstituted whole wheat flour in rats. Nutrition.

[81] Katina K. (2005). Sourdough: A Tool for the Improved Flavour, Texture and Shelf-Life of Wheat Bread, Academic Dissertation.

[82] Ross R.P., Morgan S., Hill C. (2002). Preservation and fermentation: Past, present and future. Int. J. Food Microbiol..

[83] Axel C., Roker B., Brosnan B., Zannini E., Furey A., Coffey A., Arendt E.K. (2015). Application of *Lactobacillus amylovorus* DSM19280 in gluten-free sourdough bread to improve the microbial shelf life. Food Microbiol..

[84] Alkay Z., Kilmanoğlu H., Durak M.Z. (2020). Prevention of Sourdough Bread Mould Spoliage by antifungal Lactic Acid Bacteria Fermentation. Eur. J. Sci. Technol..

[85] Rizzello C.G., Lorusso A., Montemurro M., Gobbetti M. (2016). Use of sourdough made with quinoa (*Chenopodium quinoa*) flour and autochthonous selected lactic acid bacteria for enhancing the nutritional, textural and sensory features of white bread. Food Microbiol..

[86] Sarmadi B.H., Ismail A. (2010). Antioxidative peptides from food proteins: A review. Peptides.

[87] Verni M., Verardo V., Rizzello C.G. (2019). How Fermentation Affects the Antioxidant Properties of Cereals and Legumes. Foods.

